# On the Functional Significance of the P1 and N1 Effects to Illusory Figures in the Notch Mode of Presentation

**DOI:** 10.1371/journal.pone.0003505

**Published:** 2008-10-24

**Authors:** Mathieu Brodeur, Benoît A. Bacon, Louis Renoult, Marie Prévost, Martin Lepage, J. Bruno Debruille

**Affiliations:** 1 Department of Psychiatry, Douglas Mental Health McGill University Institute, Montreal, Quebec, Canada; 2 Department of Psychology, Bishop's University, Sherbrooke, Quebec, Canada; University of Southern California, United States of America

## Abstract

The processing of Kanizsa figures have classically been studied by flashing the full “pacmen” inducers at stimulus onset. A recent study, however, has shown that it is advantageous to present illusory figures in the “notch” mode of presentation, that is by leaving the round inducers on screen at all times and by removing the inward-oriented notches delineating the illusory figure at stimulus onset. Indeed, using the notch mode of presentation, novel P1and N1 effects have been found when comparing visual potentials (VEPs) evoked by an illusory figure and the VEPs to a control figure whose onset corresponds to the removal of outward-oriented notches, which prevents their integration into one delineated form. In Experiment 1, we replicated these findings, the illusory figure was found to evoke a larger P1 and a smaller N1 than its control. In Experiment 2, real grey squares were placed over the notches so that one condition, that with inward-oriented notches, shows a large central grey square and the other condition, that with outward-oriented notches, shows four unconnected smaller grey squares. In response to these “real” figures, no P1 effect was found but a N1 effect comparable to the one obtained with illusory figures was observed. Taken together, these results suggest that the P1 effect observed with illusory figures is likely specific to the processing of the illusory features of the figures. Conversely, the fact that the N1 effect was also obtained with real figures indicates that this effect may be due to more global processes related to depth segmentation or surface/object perception.

## Introduction

**Figure 1:**
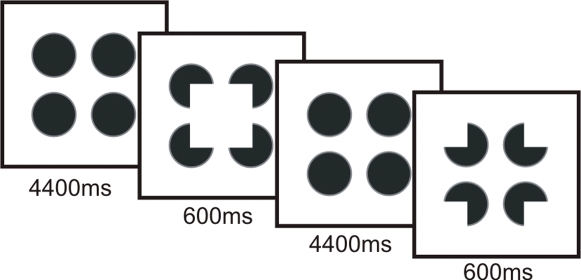
The notch mode of presentation. Notches appear at onset and disappear at offset; full black disks remain on screen between trials. In half of the trials, notches were oriented inward so as to delineate a salient illusory square. In the other half of the trials, the control condition, notches were oriented outwards.

Illusory figures evoke the vivid perception of an object in the absence of corresponding visual information. The most typical example of this type of stimulus is the Kanizsa figure, such as the one that can be seen in Figure 1. This figure is composed of four notched disks arranged in a way that generates the interpolation of an illusory square. It is generally agreed that the perception of such illusory figures depends on the emergence of illusory contours and on the modal completion of the interpolated surface [1].

Investigations of the brain mechanisms underlying the perception of illusory figures are usually conducted by extracting the brain signals elicited by the sudden appearance of the entire Kanizsa figure and by comparing them to the brain signals evoked by control figures. In this “classic” mode of presentation, both the inducers and the notches that delineate the illusory figures appear and disappear in synchrony. Recent studies, however, have shown that the perception of illusory figures could be potentiated by the use of an alternative mode of presentation in which notches and inducers are temporally dissociated [2]–[4]. In one such mode of presentation, identified as the “notch” mode, disk-shaped inducers remain on screen at all times and the notches that delineate the illusory squares appear at stimulus onset and disappear at stimulus offset.

**Figure 2:**
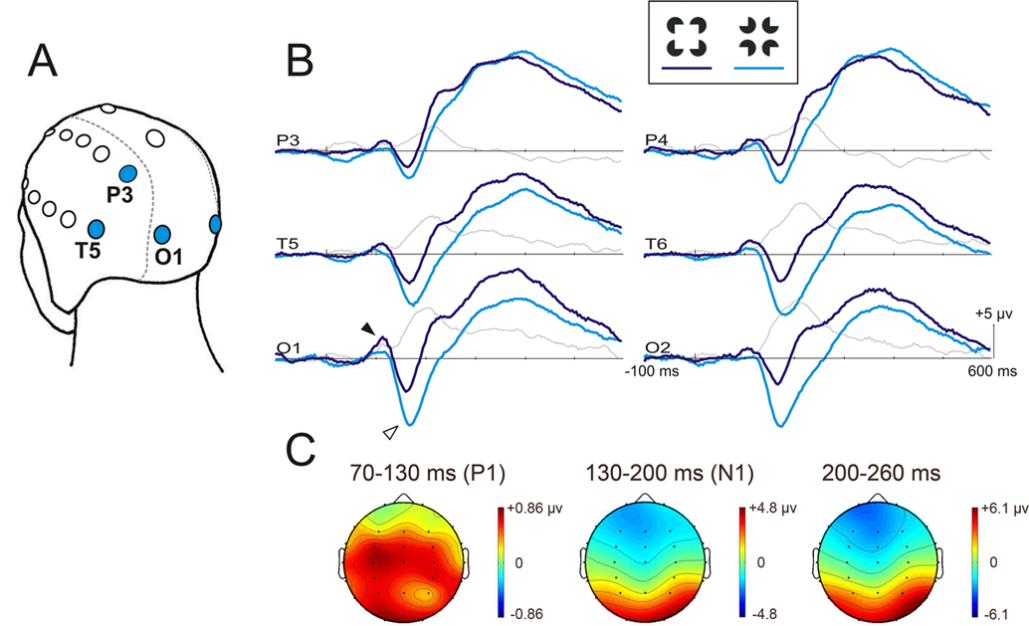
Results of Experiment 1. (A) Identification of the left-sided electrodes used in the analyses. (B) Grand averaged VEPs (n = 15) elicited by the illusory square (dark blue) and the control figure (light blue). The black arrowhead identifies the P1 and the white arrowhead, the N1. The subtraction between the amplitudes of the two VEPs is also presented (thin gray line) to illustrate the magnitudes of the figure effect across the entire epoch. (C) Mean voltage maps illustrating the topographic scalp distribution of the VEP difference (subtractions) averaged within the time-windows of 70 to 130 ms (P1), 130 to 200 ms (N1), and 200 to 260 ms.

One exciting consequence of this temporal dissociation between the notches and the inducers is that, compared to the classic mode of presentation, it reveals earlier latencies of responses to illusory contours. In a very recent visual evoked potential (VEP) study using the notch mode of presentation, we have found a modulation of the P1 that was interpreted as being related to the perception of illusory figures [2]. In the classic mode of presentation, modulations of VEPs to illusory figures can begin early but they usually peak around the N1, the negative deflection that follows the P1 [5]. The notch mode of presentation also induces an N1 modulation but in contrast to the greater N1 to illusory figures reported with the classic mode [5]–[11], the N1 is rather greater for the control figure [2].

The present study describes two experiments that aim at further exploring the P1 and N1 effects to illusory figures in the notch mode of presentation so as to clarify their functional significance. The first experiment was conducted to replicate the P1 and N1 effects to illusory figures obtained in the notch mode of presentation [2]. The second experiment was carried out with real, rather than illusory, figures. In other words, real grey squares were placed over the notches so that one condition (notches inwards) show a large central grey square and the other condition (notches outwards) show four unconnected smaller grey squares. Comparing P1 and N1 effects between Experiment 1 and Experiment 2 will clarify the functional significance of these effects. In particular, effects that are identical between conditions could be attributed to global depth segmentation or object perception processes. In contrast, effects specific to illusory figures could be directly linked to the perception of the illusory components of the displays.

## Methods

### Experiment 1

#### Participants

Fifteen healthy participants (8 females; ages ranging from 18 to 33) took part in this experiment. Participants all had normal or corrected-to-normal vision and a college level of education. They were also right-handed and they, as well as their siblings and parents, were free of neurological or psychiatric disorder. They received a compensation of 15 Canadian dollars for their participation. All participants signed an informed consent form approved by the Research Ethics Board of the Douglas Mental Health University Institute.

#### Stimuli

Four full black disks with diameters of 3.4 cm (3.3° of visual angle) permanently remained on screen. At stimulus onset, two distinct figures could appear (see Figure 1). One was an illusory square with sides of 4.4 cm (4.2° of visual angle). This square was defined by notches made in the disks. The ratio of the length of the notches' contours relative to the perimeter of the global illusory figure (i.e., the support ratio, see [12]) was 0.77. Illusory figures with such a high ratio are generally very salient [13]. The second figure was a control built from the same notched disks rotated outwards so as to disrupt the perception of the illusory square. At stimulus offset, the full black disks replaced the notched disks and remained on screen until the next stimulus onset. The disks, whether full or notched, were outlined by a gray line. This line was used by Brodeur and colleagues [2] to elaborate a condition of amodal completion. It was preserved in the present study so as to maintain the exact same stimulus conditions for purposes of comparison.

#### Procedure

The experimental design is illustrated in Figure 1. The sequence started with the appearance of the four black disks and was followed with the random presentation of 60 illusory squares and 60 control figures. Figure onset was generated by removing the portions of the disks corresponding to the notches. Offset corresponded to the restoration of the full disks. The figures appeared for 600 ms every five seconds. They were presented on a computer screen (resolution of 640×480 with a refresh rate of 75 Hz) placed 60 cm from the subject's eyes. Participants were instructed to fixate the center of the screen and to report the presence or absence of an illusory square by pressing one of two keys on the computer keyboard with their right index finger. They were also instructed to remain as still as possible and to refrain from blinking or moving their eyes during stimulus presentation.

#### Data acquisition

The recording parameters were the same as those used in Brodeur and colleagues [2]. Participants were fitted with an elastic cap of 32 electrodes disposed according to the modified expanded 10–20 system of the electrode nomenclature committee [14]. Additional electrodes were added on the right earlobe, above and below the dominant eye and at the outer canthus of both eyes. The one on the earlobe was used as the reference whereas the other four allowed the monitoring of ocular movements and eye blinks. The impedances of all electrodes were kept below 5KΩ. The EEG was recorded over 28 electrodes of the cap, which were distributed all over the scalp. The signals were amplified by Contact Precision amplifiers with a gain of 20,000. The signal was filtered with half amplitude cut-offs set at .01 and 100 Hz. An additional electronic notch filter was also used to filter the signal at 60 Hz. The EEG was sampled at 256 Hz.

#### Data measure

VEPs were extracted from EEG epochs starting 200 ms before and ending 600 ms after stimulus onset. Epochs were rejected when trials yielded an incorrect behavioural response and when the EEG of a trial was contaminated by ocular artefacts, excessive electromyogram, amplifier saturation or by analog to digital clipping as made evident by visual inspection of the data. The remaining epochs, 55 on average in each condition (±3 in the illusory condition and ±4 in the control condition), were averaged separately.

P1 and N1 amplitude were assessed relative to a −200 to 0 ms baseline. The P1 was defined as the most positive amplitude reached between 70 and 130 ms in the VEPs computed for each subject. The N1, which directly follows the P1, was defined as the most negative amplitude reached between 130 and 200 ms. These measures were specifically extracted over the electrodes that showed the greatest deflections and where the greatest figure effects occurred during the perception of illusory figures [2], [5]. These electrodes were the most posterior electrodes of the cap: O1, O2, T5, T6, P3, and P4. Reaction times and response accuracies were also recorded.

#### Data analyses

The analysis of variances (ANOVA) used to test differences over the P1 and N1 included a figure (illusory/control), and an electrode (O1, O2, T5, T6, P3, P4) factor. The Geisser and Greenhouse [15] procedure was used to compensate the heterogeneity of variance when a factor had more than two levels. The results of these analyses are reported with the original degrees of freedom, the correction factor (epsilon), and the corrected probability. Reaction times were submitted to a one-sample T-test.

As it can be seen by looking at the waveforms (Figure 2), the P1 and N1 differences were difficult to separate because they occurred in adjacent time-windows. More importantly, the VEP differences seen in the N1 time window were not limited to the N1 deflection. They were part of a larger effect that lasted until the end of the epoch. Two strategies were employed to distinguish the P1, the N1 and the subsequent VEPs. The first was used to circumvent the fact that the magnitude of the VEP effect is sometimes difficult to estimate visually, particularly over the slopes of the deflections. It consisted of the simple subtraction of the VEPs to the control figure from the VEPs to the illusory square. The second strategy was to add a time-window (2 levels) factor to the previously described ANOVA. It was conducted to verify whether the scalp distribution of the figure effect within the P1 and the N1 time-windows differed significantly. The time-windows, 70 to 130 ms for the P1 and 130 to 200 ms for the N1, were those used to identify the P1 and N1 peaks. The same analysis was conducted between the N1 and a third time-window including the averaged VEPs within 200 to 260 ms. It could thus be verified whether the figure effect occurring at the peak of the N1 was distributed over the scalp like the effect following the peak and reaching its maximal amplitude after 200 ms (see the subtractions in Figure 2).

### Experiment 2

The experiment 2 was conducted to verify whether the expected P1 and N1 effects observed in Experiment 1 were related specifically to the perception of the illusory figure or rather to a more global processes involved in depth segmentation and surface/object perception. Indeed, the notch mode of presentation is so powerful at inducing the perception of illusory contours that it may induce object perception that is qualitatively similar to that of real objects. Even in the control condition (notches outwards), the appearance of the notches may have triggered the perception of four vague illusory corners occluding the inducers in the control condition. If such perception happened, it would mean that the control condition elicited the perception of more objects than the illusory figure and this, in turn, would account for why such control condition elicited the larger N1. Experiment 2 tests this possibility by verifying whether the VEP difference observed in Experiment 1 may be replicated when real figures overlap the notches and the illusory figure.

#### Participants

Fifteen new observers (10 females, ages ranging from 19 to 30) took part in this experiment. They were recruited based on the same selection criteria as in Experiment 1 and they all signed an informed consent form approved by the Research Ethics Board of the Douglas Mental Health University Institute.

#### Stimuli and procedure

**Figure 3:**
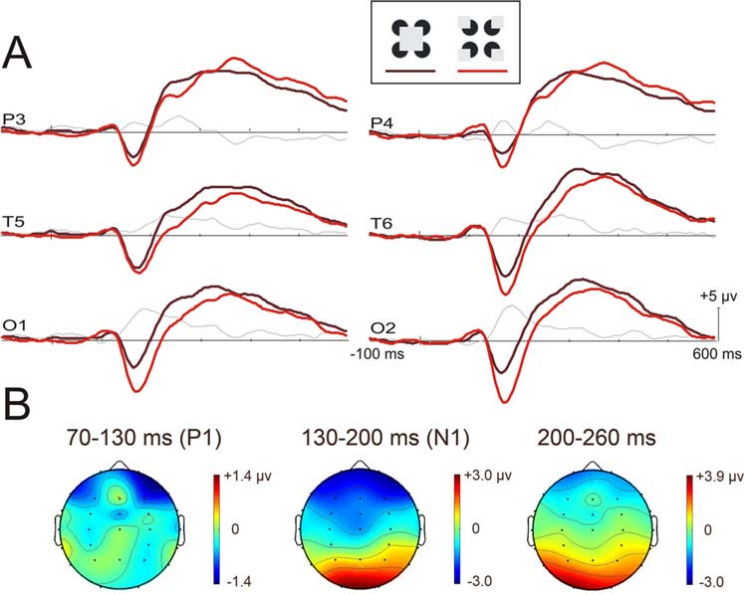
Results of Experiment 2. (A) Grand averaged VEPs (n = 15) elicited by the central square (dark red) and the four squares (light red) accompanied with the subtraction data (thin gray line). (B) Mean voltage maps illustrating the topographic scalp distribution of the VEP difference (subtractions) averaged within the time-windows of 70 to 130 ms (P1), 130 to 200 ms (N1), and 200 to 260 ms.

The stimuli were the same as in Experiment 1 except that real gray squares (mean tonal value of 229/255) were positioned so as to occlude the notches (Figure 3). In the central square figure, the real square was a perfect fit over the illusory square. In the control figure four squares, each a quarter of the illusory square surface (2.2 cm or 2.1° of visual angle), were inserted into the outward facing notches. The presentation parameters and the procedure were the same as in Experiment 1. Participants had to press on one key when the central square appeared and on another key when the four squares appeared.

#### Data measures and analyses

The VEPs were measured as in Experiment 1. The number of trials averaged after rejection was 58 for both conditions (±1 in both conditions). The analyses were however different as they needed to include the data from Experiment 1. Accordingly, we used a mixed-model ANOVA with Modality (illusory-experiment 1 vs. real-experiment 2) as a between-subjects factor and Electrodes and Figures (square vs. control) as two within-subjects factors. Because differences were observed outside the posterior region, all 28 electrodes were included in the main analysis. In case of an interaction with the electrode factor, analyses were repeated for each subset of electrodes, that is, the anterior (Fz, Fp2, Fp1, F8, F7, F4, F3), the central (Fcz, Cz, Pz, C4, C3, Cp4, Cp3, Fc4, Fc3), the temporal (Ft8, Ft7, T4, T3, Tp8, Tp7), and the posterior subset (P4, P3, T6, T5, O2, O1).

## Results

### Experiment 1

As could be expected in such an easy task, accuracy was almost perfect with correct response proportions of 99% (±2) for the illusory square and of 100% (±1) for the control figure. Reaction times were almost identical in both conditions: 594 ms (±185) and 590 ms (±182), respectively.

The grand VEPs are illustrated in Figure 2. The illusory square elicited a clear P1 and a clear N1 peaking around 106 ms and 160–170 ms, respectively. The control figure also elicited a large N1 maximum between 160–170 ms but the positive deflection in the time-window of the P1 was not well defined. The mean amplitude of the P1 was higher for the illusory square (2.7 µv ±1.7) than for the control figure (1.8 µv ±1.7). Statistical analyses indicated a figure effect (*F*(1,14) = 4.99, *p* = .042) and a nearly significant electrode×figure interaction (*F*(2,28) = 3.36, *p* = .057, ε = .872). The figure effect was more significant over occipital electrodes (O1/O2) (*F*(1,14) = 10.5, *p* = .006), than over temporal electrodes (T5/T6) (*F*(1,14) = 3.96, *p* = .066). It was not significant over parietal electrodes (P3/P4). Contrary to the P1, the mean amplitude of the N1 was greater for the control figure (−7.7 µv ±4.1) than for the illusory square (−4.0 µv ±3.0). These differences were statistically significant over all electrodes (*F*(1,14) = 26.1, *p*<.001).

Subtractions show that the P1 effect was largest at the peak of the P1, which suggests that it was a modulation of the P1. On the other hand, what we refer to as the N1 effect was maximal slightly after 200 ms, therefore more than 30 ms after the peak of the N1. Thus, this effect may index modulations of the N1 and of a later potential or it may reflect a long-lasting potential only contributing to the N1. The analyses testing the difference of scalp distribution of the P1, N1 and the 200–260 ms time-windows indicated that the P1 effect was distributed differently than the N1 effect (*F*(5,70) = 3.62, *p* = .044, ε = .361). On the other hand, the scalp distribution of the figure effect within the N1 time-window was not significantly different from the scalp distribution of the figure effect at its maximum (between 200 and 260 ms).

### Experiment 2

Again, accuracy was very high with correct response proportions of 99% in both conditions (±2 for the central square and ±3 for the four squares). Like in Experiment 1, reaction times were almost identical: 606 ms (±188) for the central square and 609 ms (±153) for the four squares.

**Figure 4:**
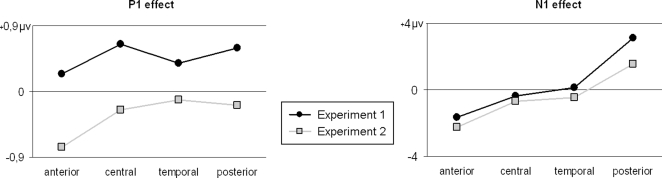
Mean amplitudes of the figure effect (illusory square vs. control figure in Experiment 1 and central square vs. peripheral squares in Experiment 2) for the four subsets of electrodes.

The VEPs to both figures presented in Figure 3 included a P1 peaking at 110 ms. The electrode×modality (*F*(27,378) = 2.51, *p*<.001, ε = .223) and modality×figure (*F*(1,14) = 11.5, *p* = .004) interactions both achieved significance, which suggests that the ERPs evoked in the two experiments were modulated differently across the scalp and that the figure effect varied depending on whether the figures were real or illusory. In fact, no P1 effect was found in the posterior region. Posterior amplitudes were indeed almost identical for the central square (2.1 µv ±1.4) and for the four squares (2.2±1.8). The only significant effect was found for the anterior subset (*F*(1,14) = 12.2, *p* = .004). Analyses with the four subsets of electrodes were also conducted on the data of Experiment 1 but no significant figure effects were found outside the posterior region. It has to be noted that the triple interaction was not significant, which suggests that the topography of the figure effect was not different across experiments. Figure 4 depicts the distribution of the effects across the four subsets of electrodes for each experiment and explains this surprising absence of interaction.

A triple interaction was obtained for the N1 (*F*(27,378) = 10.3, *p*<.001, ε = .194), meaning that the figure effect changed across experiments. Looking at Figure 4 and the scalp topographies, it can be seen that this interaction is mostly caused by a greater figure effect over the posterior region in Experiment 1. Outside this region, the modulation of the figure effect is generally similar across experiments. Just like in Experiment 1, differences of scalp distributions for the P1, N1 and 200–260 time-windows within the posterior region were tested. Results indicated that the distributions were almost significantly different between the N1 time-window and the subsequent 200–260 time-window (*F*(5,70) = 3.05, *p* = .058, ε = .435). In contrast the difference of scalp distributions of the figure effect over the P1 and the N1 was very significant (*F*(5,70) = 8.38, *p*<.001, ε = .561). To account for this difference of distribution, it must be noted that there was a small shift of about 10 ms between the peak of the figure effect (see the subtraction data in Figure 3) and the N1 at the occipital electrodes. This shift was smaller than the corresponding shift observed in Experiment 1. Shifts of latency frequently introduce changes of amplitude and as such, the small shift in Experiment 2 may explain why amplitude over posterior region was smaller in Experiment 2 than in Experiment 1.

N1 amplitude difference was significant in the anterior (*F*(1,14) = 21.2, *p*<.001) and posterior subsets (*F*(1,14) = 8.06, *p* = .013). The greater N1 effect, that is the most negative amplitude within the N1 time-window, was found in the posterior subset and more specifically at occipital electrodes as suggested by the significant figure×electrode (of this subset) interaction (*F*(2,28) = 6.27, *p* = .006, ε = .971). In this subset, the N1 was larger for the four small outwards squares (−7.6 µv ±5.8) than for the large central square (−5.3 µv ±4.4) condition. The analyses testing for a figure effect in Experiment 1 showed that in addition to the posterior effect reported previously, there was also a figure effect in the anterior subset (*F*(1,14) = 11.9, *p* = .004).

## Discussion

The present study investigated the functional significance of the P1 and N1 effects induced by the perception of illusory figures in the notch mode of presentation. The VEP differences between figures in Experiment 1 were successful replications of those reported in Brodeur and colleagues [2]. The P1 and the N1 evoked by the illusory figure were respectively larger and smaller than the P1 and N1 evoked by the control figure. In Brodeur and colleagues [2], other figures were also presented in separate conditions but the present results showed they were not necessary for the P1 and N1 effects to occur. Subtractions and scalp distribution analyses provide further details on the P1 and N1 effect. First, as illustrated in Figure 2 (see panel C), these two effects have their own scalp distributions and are therefore unlikely to be part of a unique broad effect covering the two deflections [16]. Also, it is noteworthy that what we refer to as the N1 effect was not centered over the N1 deflection. The figure effect started with the N1 but it apparently peaked later, after 200 ms. The scalp distribution analysis suggested that the figure effect occurring within the time-windows of the N1 and after the N1 is the same. It however cannot be thrust aside that this broad effect is made of two potentials that add their influence around 200 ms but that have analogous generators, given the similarity of the scalp distributions (Figure 2C).

The second experiment was conducted to differentiate between VEP effects specifically due to the illusory nature of the figure and those linked to normal, global processes related to the segmentation and perception of a surface or object. To do so, VEPs were recorded in response to real grey squares superposed over the notches at stimulus onset. When the notches were turned inwards, the perceptual result was a large central grey square. When the notches were turned outwards, four little grey squares stood in them. We found no P1 effect over the posterior region. However, as the global scalp distribution of the effect was not significantly different across experiments (see figure 4), we can assume that the generators involved in the effect were the same. The real figures of Experiment 2 nevertheless elicited a modulation in the frontal region that was not observed in Experiment 1, as if a global shift of amplitude pulled out the ERPs toward the negative polarity, therefore causing the cancellation of the posterior (positive) effect and the emergence of a negativity over the anterior region. The mechanisms solicited in the two experiments might therefore have been activated to different extents across the brain. The lack of a posterior P1 effect in Experiment 2 combined with the likelihood of comparable generators across experiments strengthens the idea that the P1 effect is specific to the processing of illusory contours. Further testing and analyses allowing for a more precise definition of the generators may however be needed to confirm the common origin of the P1 elicited in the two experiments. It also should be noted that the between-subjects design used herein may have limited the comparison between the results of Experiment 1 and Experiment 2.

The N1 effect was also compared between illusory and real figures. In both experiments, the amplitude within the N1 time-window is larger for the control condition and the scalp distribution of this modulation within the posterior subset is different from the scalp distribution of the P1 effects. It has to be noted that the N1 effect was not restricted to a posterior modulation but also included an anterior modulation. The only difference is that the N1 effect ended earlier in Experiment 2 but this may be due to the fact that figures were real and, therefore, may not require as much top-down modulation as illusory figures [17]. This difference of latencies attenuates the amplitude of the posterior N1 effect but not the anterior effect and that may account for the variation of the figure effect across experiments. As hypothesized, the greater number of squares and probably also the greater complexity of the four square condition can be advanced as possible causes for the greater N1 elicited in Experiment 2. Following this reasoning, the greater N1 in response to the control figure than to the illusory square in Experiment 1 could likely be due to the processing of four illusory squares.

### 

#### P1 effect

In accordance with the general agreement as to the functional significance of the P1, it can be argued that the P1 effect reflects a greater or deeper processing of contour and form information in the illusory square condition. Pioneer investigations of the P1 showed that the amplitude of this deflection is modulated by spatial frequencies and contrast, two variables intrinsically linked to the perception of contours [18], [19]. These observations were generally obtained using checkerboards and gratings but the P1 has since been shown to also vary in amplitude as a function of more stimulus complexity [20], [21]. In Brodeur and colleagues [2], the best dipole fitting located the P1 generators in the fusiform gyrus. The fusiform gyrus is a structure known for its essential role in the processing of objects [22], [23] even when these are illusory [24], [25].

To our knowledge, P1 effects similar to the one elicited by the notch mode of presentation in Brodeur and colleagues [2] and in the present study have never been reported in the literature. The only study reporting a P1 effect to an illusion that may be related to the perception of illusory figures was conducted by Hayashi and colleagues [26]. Their observers were presented with two checkerboards that contained the same amount of physical information; one induced a distortion illusion and the other did not. The P1 to the illusion was greater than the P1 to the control, which again suggests that the neural mechanisms involved in illusory changes of contours occurred over this deflection.

The enhanced processing of the illusory square, as reflected by the larger P1, can only be accounted for by the processing of illusory-related features given that the same amount of real information were present in the illusory and in the control condition. Here, we assume that these features are primarily the illusory contours but they could also be related to the enhanced brightness of the illusory figure which is also known to modulate the P1 amplitude [27], [28]. Spatial attention, another variable known to affect the P1, remains however a potential confound that is not directly related to the processing of illusory figures [29]. A stimulus appearing over a covertly attended area indeed elicits a P1 of greater amplitude than a P1 appearing over an unattended area [30]–[32]. This modulation is generally accounted for by a gain in the stimulus processing signal [33]. The illusory square could have benefited more from attention since the area it covered was central, relatively small, uninterrupted, and therefore more likely to be within the attended space than the control figure. Moreover, although subjects had to behaviourally respond to both types of figures, they might have been more prompt to use the presence of the square and consequently, the space that it delineated, as the main discrimination criterion. Accordingly, the greater P1 could simply reflect a gain in the processing of the more attended notches in the illusory square. However, this possibility was seriously undermined by Brodeur and colleagues [2] who reported that the P1 to the illusory square was not only greater than the P1 to the control figure but also than the P1 to an amodal square. The notches of this square covered the same spatial area as those of the illusory square but they could not induce illusory contours. Therefore, if spatial attention did provide a gain on a signal, it could only have done so on the signal triggered by the illusory contours. The greater P1 to the illusory square relative to the amodal square is also noteworthy as it shows that the P1 effect was not simply consequent to the perception of a square independently from the illusory contours. The contours of the notches are obviously sufficient to trigger the perceptual mechanisms leading to the representation and the recognition of the square. Our previous results with the amodal square clearly suggest that the P1 modulation also involves mechanisms that are specific to the illusory induction [2].

Although it represents a more parsimonious and more powerful way of presenting illusory stimuli, the notch mode of presentation has not yet been widely used. A result common to all the experiments that used the notch mode is that illusory figures seem to be more salient and to be accompanied by earlier brain activities [2], [3] and by the greater visual potency of the figure [34]–[36]. The notch mode of presentation has also been shown to induce responses from cells located in more primary cortices of the monkey brain than the classic mode. Indeed, in the classic mode of presentation, cells responsive to illusory contours induced by Kanizsa-like stimuli have systematically been reported in V2 [37]–[39] (see [40] for a review) but not in V1. Using the notch mode of presentation, Lee and Nguyen [3] succeeded at finding such cells in V1, at the border of V2. In addition, Davis and Driver [34] showed that the detection of an illusory figure appearing in the notch mode of presentation is automatically achieved. In contrast, in the classic mode, detection is usually serial, as suggested by an increase of the detection time as the number of distractors increases [41]–[43].

The beneficial influence of the notch mode of presentation could also be present in other modes of presentation where the binding of separate elements is facilitated. For instance, presenting illusory figures as stereograms significantly increases saliency [44]–[46] and the related neural activities [47]. Likewise, moving together the notches from one set of inducers to another set also promotes visual binding, mostly because, as in the notch mode of presentation, the notches share a common temporal feature (i.e., their movement) [48]. For example, Seghier and colleagues [4] used motion to evoke very early human brain responses to illusory figures, in V1, much earlier than the usual extrastriate activities evoked by static illusory figures. Additionally, it has been shown that motion allows the perception of illusory figures in 3–4 month-old infants and even in newborns. Static figures, by comparison, can only be perceived from 6 to 8 months of age [49]–[51]. In fact, it may not be the classic mode of presentation per se that prevents the emergence of a P1 effect. The illusory figures viewed through this mode of presentation may simply not be salient enough to strongly activate these early neural mechanisms. Accordingly, by using more salient illusory figures, one could expect to find a P1 effect even in the classic mode of presentation. This hypothesis however remains to be verified.

The facilitation provided by the modes of presentation that promote binding does not operate only for illusory figures but also for incomplete or fragmented stimuli that induce no illusory contours [3], [39], [43], [52]–[55]. For instance, the invisible portion of an amodal line can trigger responses in macaque V1 neurons when the obstacle is stereoscopically placed in front of the inducing line segments [39], [53]. These responses do not occur without stereoscopy or when the disparity relationship is inverted. In a similar vein, amodal contours presented according to the notch mode of presentation elicit a slight response in V1 while control figures do not [3]. As a final example, Kellman and Spelke [54] showed that motion contributes to lower the age at which infants become able to perceive amodal figures, just like it does for illusory figures. Could this mean that the P1 effect reported herein indexes a binding process that operates independently from the formation of illusory contours? This is unlikely given that the P1 observed in Brodeur and colleagues [2] and the V1 response shown by Lee and Nguyen [3] were still larger for the illusory figure than for the amodal figure.

#### N1 effect

At first glance, the fact that the control figure evoked a greater N1 than the illusory figure in Experiment 1 goes against the generalized assumption that spatio-temporal modulations occurring within the time range of the N1 reflect the object processing of the illusory figure [5], [20], [56]. Usually, the object-selective N1 (and activation in its associated brain area, the lateral occipital complex) is found to be smaller for fragments that cannot be bound and larger for fragments, segments of lines, gabors (cosine patches within a gaussian window) or notches that can be brought together as a global object with or without illusory contours [56]–[60]. However, the present results may not be as contrary to the literature as they appear. Indeed, the control figure may simply not be acting as a control because the notch mode of presentation provides a cue of segregation (see [61] for a review on these cues) that is powerful enough to induce a more complex perception than that induced by the classic mode of presentation. In accordance with the object-selective account of N1, this complex perception could involve four objects that seem to overlap the inducers when the notches are turned outwards. Larger N1 to the control than to the illusory figure could thus be accounted for by this greater number of objects perceived (i.e. four versus one).

One issue that arises then is whether the four occluding corners are illusorily prolonged as if they belonged to undefined forms. The fact that no illusory contours were perceived in the control condition argues against this possibility. Nevertheless, the lack of such perception may be due to the fact that, for each object, there was only one inducer whereas it is usually assumed that two inducers are required for illusory contours to be perceived. Grossberg [62], in his LAMINART model, argued that neurons coding for the illusory contours in layers 2/3 of the visual cortices exhibit the property of bipole grouping. This property is implemented by the balance of the excitatory long-range interactions between neurons coding for neighbouring receptive fields on the one hand, and of the inhibitory action of short-range interneurons on the other. A single inducer activates neurons coding for illusory contours but, at the same time, it activates interneurons that inhibit those neurons. The end result is that the neurons that code illusory contours remain unresponsive. In situations where two inducers are present, the interneurons inhibit each other and this allows for the summation of the excitatory activities of the neurons that code for illusory contours. Nevertheless, the inability of a single inducer to elicit illusory contours does not mean that it is unable to activate processes specific to object perception. It only suggests that if these processes are activated, their output remains under the threshold of consciousness. Dresp and Bonnet [63] reported an intriguing result that tends to support this possibility. Their participants were required to detect a small light spot. Detection was much easier when the spot was presented over an illusory contour. Surprisingly, this facilitation was also observed when the spot was presented over the extension of a single inducer contour. The authors concluded that a single inducer, like the unconnected inducers of our control figure, can provide sufficient local information to trigger the basic neural mechanisms that can *potentially* induce the perception of an illusory figure.

The question however remains as to the nature of the local information at stake in the tendency to produce illusory contours. Interestingly, the search for this information provides an account of the N1 effect different from that of the greater object processing, although both accounts are not at all mutually exclusive. The local information could be the one that leads to four separate depth segmentations for the control figure. Segmentation occurs locally when a portion of the stimuli contains sufficient information to determine the relative depth position of the surface separated by the contours. The T-junction is a typical example of a local depth segmentation cue or interposition cue. Without having to analyze the whole stimuli, it can be determined that the surface above the horizontal bar of the T is placed over the surface below this horizontal bar. As early as 1972, Coren [64] argued that notches represent superposition cues that are processed independently from the global perception of illusory figures (see also [65] for details on local depth cues). In other words, an inducer by itself contains sufficient information to determine that something overlays a disk and it is this information that, in turn, allows for the emergence of the illusory figure. Experimental demonstrations support this assumption [66]–[68] and theoretical models including a concept of border ownership have suggested that figure-ground organization can be encoded along with contour processing [69], [70]. This concept of border ownership has recently been supported by single-cell recordings in monkeys showing that the response of cells coding for contours is modulated by the figure-ground relationship of the two sides separated by the contour [47], [71], [72].

Strong local depth segmentation could be responsible for the potentiation provided by the notch mode of presentation and for the greater N1 evoked by the control figure. In the classic mode of presentation, these depth cues would simply not be sufficiently salient to induce a large N1. The larger negativities found within the time-range of the N1 for the control figure in the notch mode of presentation are very similar to those evoked by segregation and depth processing. For example, an array of radially expanding elements eliciting the illusory perception of perspective evokes an enlarged LNP, a negative deflection peaking around 200 ms [73]. With illusory figures, it has also been shown that inducers, which present no apparent local cues, elicit a smaller N1 than notched inducers [74]. Like the modulation induced herein by the control figure, the modulations induced by depth segmentation are not centered on the N1 but slightly delayed and maximal at occipital electrodes. The modulation of VEPs within the time-range of the N1 by the control figure also recalls the texture segmentation VEP (tsVEP) that is computed by subtracting the VEPs to a pattern of lines arranged so as to define a form or a checkerboard from the VEPs to a uniform pattern of lines [75]. Although debatable, tsVEP has often been proposed to reflect a figure-ground segmentation or surface processing [76], [77]. The tsVEP is generally indexed by a posterior negativity occurring between 161 and 225 ms and is known to increase with the number of segregation cues or with the saliency of the segregation [78], [79]. Note that, just like the modulations induced by depth segmentation, the tsVEP peaks slightly later than the N1. Moreover, cells that underlie analogous functions to the tsVEP generators have been reported in V1 cortex of monkeys [80].

It might appear incongruent to associate depth segmentation with a neural signal (i.e., the N1) that follows the neural correlate found for illusory contours perception (i.e., the P1). However, it is not incongruent if one takes the following two assumptions into account.

The first assumption is that the influence of depth segmentation on the perception of illusory contours is modulatory and not causal. The existence of descending projections from object-processing visual areas is well established [5], [17] as is the modulatory capacity of different cognitive factors on the saliency of illusory contours [81]–[83]. A convincing demonstration of the late influence of depth segmentation on the saliency of illusory contours has been provided by Reynolds [84]. This author elaborated an illusory figure and added bars that passed over the illusory area but under the inducers. These bars had an important weakening effect on the illusory figure because they acted as conflicted depth cues, placing the illusory figure under the inducers. This figure was then presented to observers and masked after variable time intervals (SOAs) so as to interrupt visual processing. The majority of participants reported illusory contours when SOA was 150 ms but they lost this perception when SOA was 300 ms. This experiment suggests that illusory contours are elaborated before depth information can interfere with them. The second assumption is that contours and depth information are processed relatively independently [85]–[88]. Indeed, depth processing can occur locally, without the perception and the interpretation of the global figure. Accordingly, in the contour processing system, the illusory contours would first be induced, although at an intermediate level of saliency. Meanwhile, the global figure would be interpolated probably with the contribution of the illusory contours [6]. In parallel, the local depth cues would be extracted on the basis of primary contour processing and combined with the globally interpolated figure. This combination would provide the necessary elements for a cognitive interpretation of the figure which would then be followed by modulatory top-down influences by which some contours (particularly the illusory ones) would be enhanced while others would be suppressed [86].

The following methodological limitations have to be taken into consideration for future investigations. Participants in Experiments 1 and 2 were different (between-subjects design), which lowered the statistical power of the analyses of the effect of the modality factor. Statistical power was however high enough to obtain a significant interaction involving the between-subject factor. The fact nevertheless remains that although there was clearly an absence of P1 effect in Experiment 2, it cannot be concluded without the shadow of a doubt that the same participants would necessarily exhibit a P1 effect with the illusory figures of Experiment 1. In addition, one could argue that illusory figures are more intriguing and consequently more catchy than real figures. This difference between the types of figures can potentially induce a CNV-like difference in the pre-stimulus period which in turn, would differentially affect the post-stimulus ERPs. However, the greater the CNV, the more important the positivity to return to the baseline. Accordingly, the ERPs of experiment 1 should include a positive shift when compared to the ERPs of experiment 2. This was not the case. Thus, there may be no CNV problem.
